# Large-scale networks changes in Wilson’s disease associated with neuropsychiatric impairments: a resting-state functional magnetic resonance imaging study

**DOI:** 10.1186/s12888-023-05236-3

**Published:** 2023-11-03

**Authors:** Anqin Wang, Ting Dong, Taohua Wei, Hongli Wu, Yulong Yang, Yufeng Ding, Chuanfu Li, Wenming Yang

**Affiliations:** 1 The First Affiliated Hospital of Anhui University of Chinese Medicine, Hefei, 230031 Anhui China; 2 Xin ‘an Institute of Medicine and Modernization of Traditional Chinese Medicine, Institute of Great Health, Hefei National Science Center, Hefei, China; 3 Key Laboratory of Xin’An Medicine, Ministry of Education, Hefei, China; 4 School of Medical Information Engineering, Anhui University of Chinese Medicine, Hefei, 230012 Anhui China

**Keywords:** Wilson’s disease, Independent component analysis, Resting-state networks

## Abstract

**Background:**

In Wilson’s disease (WD) patients, network connections across the brain are disrupted, affecting multidomain function. However, the details of this neuropathophysiological mechanism remain unclear due to the rarity of WD. In this study, we aimed to investigate alterations in brain network connectivity at the whole-brain level (both intra- and inter-network) in WD patients through independent component analysis (ICA) and the relationship between alterations in these brain network functional connections (FCs) and clinical neuropsychiatric features to understand the underlying pathophysiological and central compensatory mechanisms.

**Methods:**

Eighty-five patients with WD and age- and sex-matched 85 healthy control (HC) were recruited for resting-state functional magnetic resonance imaging (rs-fMRI) scanning. We extracted the resting-state networks (RSNs) using the ICA method, analyzed the changes of FC in these networks and the correlation between alterations in FCs and clinical neuropsychiatric features.

**Results:**

Compared with HC, WD showed widespread lower connectivity within RSNs, involving default mode network (DMN), frontoparietal network (FPN), somatomotor network (SMN), dorsal attention network (DAN), especially in patients with abnormal UWDRS scores. Furthermore, the decreased FCs in the left medial prefrontal cortex (L_ MPFC), left anterior cingulate gyrus (L_ACC), precuneus (PCUN)within DMN were negatively correlated with the Unified Wilson’s Disease Rating Scale-neurological characteristic examination (UWDRS-N), and the decreased FCs in the L_MPFC, PCUN within DMN were negatively correlated with the Unified Wilson’s Disease Rating Scale-psychiatric symptoms examination (UWDRS-P). We additionally discovered that the patients with WD exhibited significantly stronger FC between the FPN and DMN, between the DAN and DMN, and between the FPN and DAN compared to HC.

**Conclusions:**

We have provided evidence that WD is a disease with widespread dysfunctional connectivity in resting networks in brain, leading to neurological features and psychiatric symptoms (e.g. higher-order cognitive control and motor control impairments). The alter intra- and inter-network in the brain may be the neural underpinnings for the neuropathological symptoms and the process of injury compensation in WD patients.

## Introduction

WD is a monogenic inherited autosomal recessive disorder caused by ATP7B gene mutation, resulting in defective copper metabolism and gradual accumulation of the copper in body organs, especially the basal ganglia in the brain [1, 2]. Neuronal injury caused by excessive copper build-up included central pontine myelinolysis, demyelination, astrogliosis, abnormal iron deposition and cavitation, leading to neurological and neuropsychiatric symptoms [3–6]. Typical neurological symptoms present as the initial appearance of one or a group of symptoms, usually manifested as speech disorders, tremors, stiffness, gait disturbances, and decreased cognitive abilities. Seizures, myoclonus, and pyramidal tract signs may also occur [7, 8]. There are several studies that have been conducted on this topic. Research has demonstrated a positive correlation between the concentration of sNfL and scores indicating the severity of neurological conditions, as well as acute and chronic brain damage as determined by the neuroimaging scale [9, 10]. Psychiatric symptoms are prevalent in approximately one-third of WD patients. At the onset of the disease, the most frequently observed symptoms include personality change, irritability, anxiety, and depression. However, as the disease advances, additional manifestations such as impulsivity, disinhibition, catatonia, and mania can arise. Moreover, reckless behavior may also be exhibited by affected individuals [7]. Penicillamine and trientine were used to facilitate the urinary excretion of copper for ‘de-copper’ patients, to decelerate the progression of the deterioration [5]. The majority of appropriately treated patients experience positive outcomes, including improvements in liver function tests and neurological deficits. Nevertheless, partial individuals may continue to experience persistent or worsening neurological symptoms [11, 12].

To explore the underlying neurofunctional mechanisms of brain damage in WD patients, numerous neuroimaging studies have been conducted, documenting both structural and pathological brain abnormalities [13–15]. Shribman [13] observed that the severity of neurological deficits correlated with grey matter volume in predominantly subcortical regions, with a decrease in axial diffusivity in white matter tracts, and with iron deposition in widespread cortical regions in chronically treated patients, via a multimodal, whole-brain MRI study. In addition, numerous studies have documented distinct levels of functional connectivity changes in the brains of WD patients [16, 17]. The brain is known to be a highly interconnected complex network of neurons, with several functional sub-networks interacting with one another to sustain the activity of the entire brain [18]. Complex neurological symptoms are brought on by changes in the corresponding sub-networks brought on by localized lesions, which altered the effectiveness of total brain activity [19, 20]. Han [21] discovered that the alerting function in particular in an attention network test revealed poor attention functions in WD patients. Han conducted a follow-up study in which, using tools for graph-theoretic functional brain network analysis using the posterior cingulate cortex (PCC) as a seed, he looked at the DMN. Attention dysfunction was associated with functional connection between the left inferior temporal cortex and the right lateral parietal cortex, and WD patients were found to have lower local and global network efficiency than healthy controls [22]. Still, current research has not yet involved the sub-networks composed of multiple brain regions and the mechanisms of synergy between the networks in WD patients.

The ICA, a data-driven and multivariate approach that makes no prior assumptions, has been shown to be a useful tool for detecting and isolating different brain function networks [23–25]. With the ICA approach used to analyse fMRI data, the spatial independent components (ICs) and the corresponding mixing matrix could be obtained, and the latter could be used to calculate functional connectivity between RSNs [25]. In brain mapping, nodes represent functionally independent RSNs and edges represent functional connections between RSNs. In this study, we aimed to investigate alterations in brain network connectivity at the whole-brain level (both intra- and inter-network) in WD patients through ICA. Furthermore, this study explores the relationship between alterations in these brain network FCs and clinical neuropsychiatric features to understand the underlying pathophysiological and central compensatory mechanisms.

## Materials and methods

### Subjects

This study was approved by the ethics committee of the Institutes of the First Affiliated Hospital of Anhui University of Traditional Chinese Medicine before the beginning of the study. All methods were performed in accordance with the relevant guidelines and regulations. All participants have signed the informed consent form before participating in the study procedures. Eighty-five patients with WD treated in the neurology department of our hospital in 2021 were included in this study. Inclusion criteria: (1) patients with diagnosis of WD according to the Leipzig diagnostic criteria were included [26], based on the clinical neuropsychiatric symptoms, low serum ceruloplasmin lever, decreased activity of copper-dependent oxidase, Kayser-Fleischer (KF) ring and increased 24-h urinary excretion of copper; (2) age 14 years or older; (3) without other neuropsychiatric disorders; (4) right-handed. Exclusion criteria: (1) with additional neurological or psychiatric disorders; (2) using neurologic or psychotropic drugs; (3) patients with contraindications to MRI such as claustrophobia or pacemakers; (4) non right-handed. Thus, 85 patients (53 males and 32 females) were included for the follow-up analysis. Clinical and biochemical data of patients with WD were recorded, including the UWDRS-N, UWDRS-P, serum ceruloplasmin (SC), 24-h urinary copper (24-h UC), and course of disease (DC). For the controls, age- and sex-matched 85 healthy subjects from social officers had been recruited (49 men and 36 women). The equal exclusion standards have been adopted as the patient group. Detailed records about the participates are shown in Table 1.

**Table 1:** Lists the clinical and biochemical data of WD patients

At initial presentation	Patients of WD	Controls
Patients (NO.)	85	85
Males/Females (NO.)	53/32	49/36
Ages (years, mean ± SD)	27.40 ± 8.05	25.83 ± 5.50
UWDRS-N score	4.01 ± 4.47	/
UWDRS-P score	1.98 ± 1.88	/
24 h urinary excretion of copper ($\upmu$ mol/L)	824.45 ± 503.63	/
course of disease (years, mean ± SD)	8.8 ± 6.08	/
serum ceruloplasmin (g/L)	0.05 ± 0.04	

### Image acquisition

The entire brain was covered in images that were acquired in a line parallel to the anterior–posterior commissure line on a 3.0 T-MRI scanner (Discovery MR750, GE Healthcare, Milwaukee, WI, USA), with an 8-channel radiofrequency coil. Foam padding was employed to immobilize the subjects' heads during image acquisition to minimize movement, and earplugs were used to minimize scanner noise. Subjects were instructed to relax, keep their eyes closed, and remain sober while scanning. The parameters of the resting-state functional image sequence were as follows: repetition time (TR): 2000 ms; echo time (TE): 30 ms; slices; 36; thickness: 3 mm; gap: 1 mm; field of view (FOV): 220 mm × 220 mm; acquisition matrix: 64 × 64; and flip angle (FA): 90°. The sagittal high-resolution T1-weighted images were acquired with TR: 8.2 ms; TE: 3.2 ms; slices: 166; thicknes: 1 mm; gap: 0 mm; FOV: 256 mm × 256 mm; acquisition matrix: 256 × 256; and FA: 12°. We checked all images to ensure that the images used in the subsequent analysis were free of artifacts or head movements.

### MRI data preprocessing

The resting-state fMRI data preprocessing was carried out using Data Analysis for Brain Imaging software (DPABI, version 6.0, http://rfmri.org/DPABI [27], based on Statistical Parametric Mapping 12 implemented in MATLAB (version R2016b; MathWorks, Natick, MA). The following procedures were used to pre-process the data: (1) original DICOM pictures were first converted to NIfTI format; (2) the first 10 time points of each subject were removed, to allow for the steady-state of magnetization and the patient's acclimatization to the scanning environment; (3) the rest of 175 time points were slice timing corrected and realigned for head-motion correction, to make sure that the image acquisition time was consistent amongst slices and to avoid with the impact of movement on FC. Participants with a maximum displacement of 2 mm and a maximum rotation of more than 2° were excluded for further analyses. (4) co-registration of functional images with individual T1-weighted images and spatial normalization to the Montreal Neurological Institute (MNI) template with a resampling voxel dimension of 3 mm × 3 mm × 3 mm, using a nonlinear registration tool of Diffeomorphic Anatomical Registration Through Exponentiated Lie algebra (DARTEL) [28]; (5) spatially smoothed using a Gaussian kernel of 6 mm full width at half maximum to reduce registration errors.

### Independent component analysis

#### Identification of resting-state networks

Using ICA, a data-driven, multivariate method without priori assumptions, unknown mixed fMRI signal sources might be divided into maximal spatial activation maps or independent temporal components. In order to define RSN components, analyses of this work were carried out using the ICA with GIFT program (http://icatb.sourceforge.net, version 3.0c). 1) The group ICA approach was used to concatenate individual data across time and subsequently compute subject-specific components and time-courses; 2) Data reduction was performed with principal component analysis (PCA), and the number of ICs was estimated with the minimum description length. 3) To ensure data repeatability, the Infomax algorithm was used to extract independent spatial maps and time-courses for each component, and the ICASSO was performed 100 times [29]. 4) The individual time courses and spatial maps were received using the group ICA back-reconstruction. After the reverse reconstruction, the individual level of time course IC and space IC maps was converted into a z-score. The z-value reflects the FC's strength.

#### Intra-network FC analysis

After defining the RSNs, we applied one sample t-test on the corresponding z maps of ICs from two groups and took the sum of them to create masks, using a voxel-level family-wise error (FWE, *p* < 0.05) correction with a cluster size of > 100 voxels. Subsequently, a two- sample t-test was performed on the z maps of each RSN within the masks, with age and gender as covariates for regression, to examine the regional differences in the networks between groups. Results were corrected for multiple comparisons using the cluster-level FWE method, with a voxel *p* < 0.001 and a correction threshold of *p* < 0.05. The FC intensity of individual-level clusters with a significant group difference in each network was extracted, and visualized with brain regions and violin maps, and compare the differences in FC between WD patients with abnormal UWDRS-N / UWDRS-P scores and those with normal scores.

#### Inter-network FC analysis

The MANCOCAN toolbox implanted in the GIFT (Version 3.0c) software was employed to obtain relationships between RSNs. Temporal band-pass filtering (0.1–0.15 Hz) of the imaging data used to be conducted to minimize the effects of low-frequency drift and high-frequency physiological noise. Then the correlations between any two RSN time courses of each subject were calculated. Pearson's correlation coefficients (r) were calculated for the mean time-courses of RSNs and then transformed to z-values using Fisher's Z-transformation. Accordingly, a 12 × 12 matrix was obtained for further between-group analyses. Finally, a two-sample t-test was used to examine the group differences between HC and WD for each pair of RSNs; age and gender were considered as covariates for regression (*p* < 0.05, with the false discovery rate (FDR) corrected). The mean z scores of inter-network connections with significant differences were extracted using MATLAB software for further correlations analysis.

### Statistical analyses

Statistical Package for Social Science software (SPSS, v27.0, Chicago, IL, USA) was used to investigate differences between groups, with a P-value of 0.05 considered statistically significant, independent two-sample t-tests for continuous variables and χ2 test tests for categorical variables. Pearson’s correlation analysis was used to assess the correlations between clinical and biochemical characteristics and altered intra- and inter-network FCs in the patient group, including UWDRS-N score, UWDRS-P score, age, DC, SC, and 24-h UC, with a 0.05 significance level adopted. Cohen’s d was then used to describe the effect size of intra-network FCs Z-Value.

## Results

### Demographic and clinical information

The demographic and clinical characteristics of the WD patients and controls are summarized in Table 1. There were no differences in age and gender distribution between WD and HC.

### Components of the resting-state networks

**Fig. 1:**
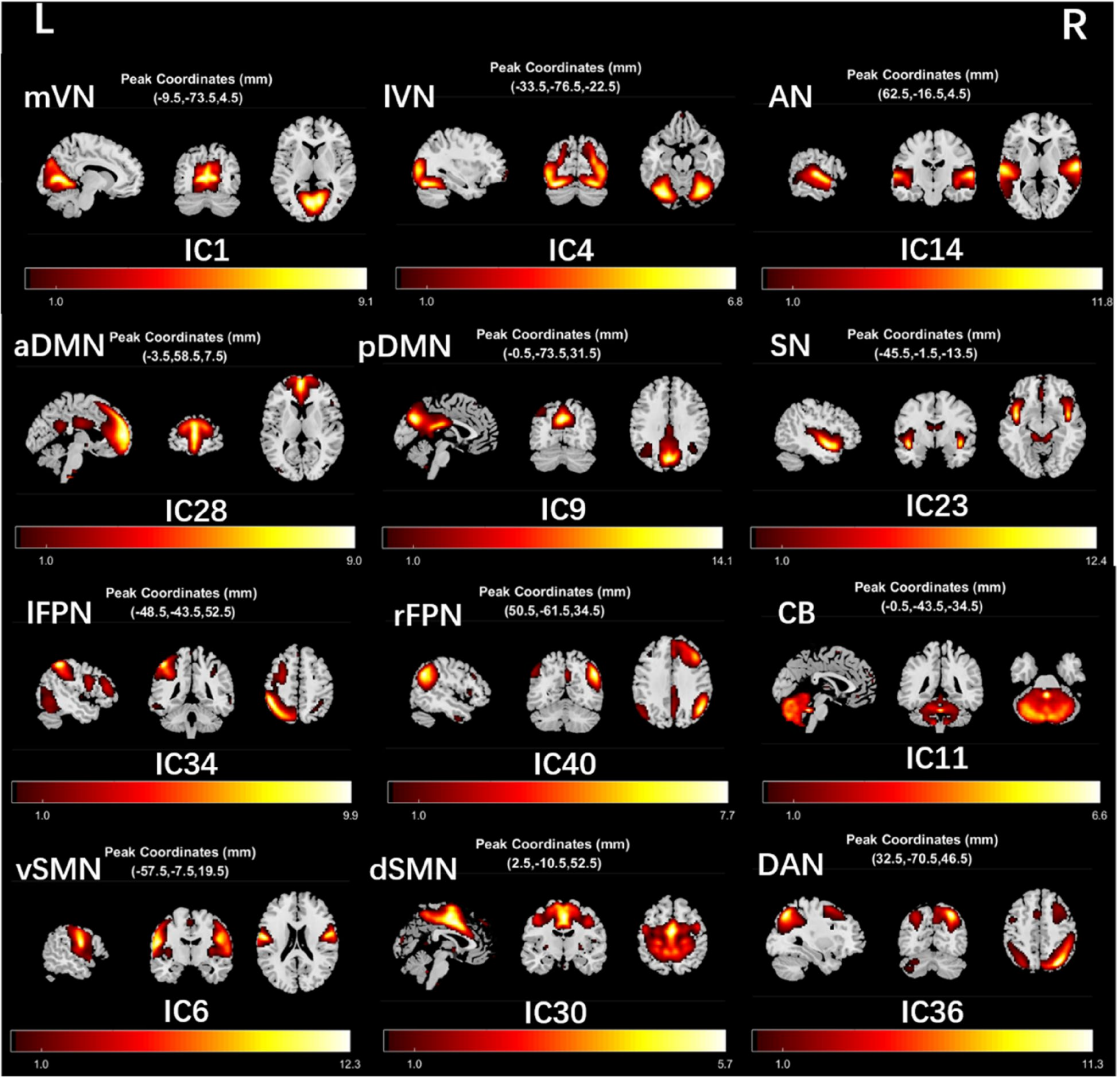
Components of the RSNs. Abbreviations: mVN: medial visual network; lVN: lateral visual network; AN: auditory network; aDMN: anterior default mode network; pDMN: posterior default mode network; SN: salience network; lFPN: left frontoparietal network; rFPN: right frontoparietal network; CB: cerebellum network; vSMN: ventral somatomotor network; dSMN: dorsal somatomotor network; DAN: dorsal attention network. The color bar indicates the T-value

Among the components arising from ICA, 12 meaningful RSNs were identified as the focus of subsequent analysis via visual inspection (Fig. 1), and these RSNs were consistent with previous rs-fMRI studies [30–33]. The RSNs comprised medial visual network (mVN, IC1), lateral visual network (lVN, IC4), auditory network (AN, IC14), anterior default mode network (aDMN, IC28), posterior default mode network (pDMN, IC9), salience network (SN, IC23), left frontoparietal network (lFPN, IC34), right frontoparietal network (rFPN, IC40), cerebellum network (CB, IC11), ventral somatomotor network (vSMN, IC6), dorsal somatomotor network (dSMN, IC30), dorsal attention network (DAN, IC36).

### Aberrant intra-network FC

**Fig. 2:**
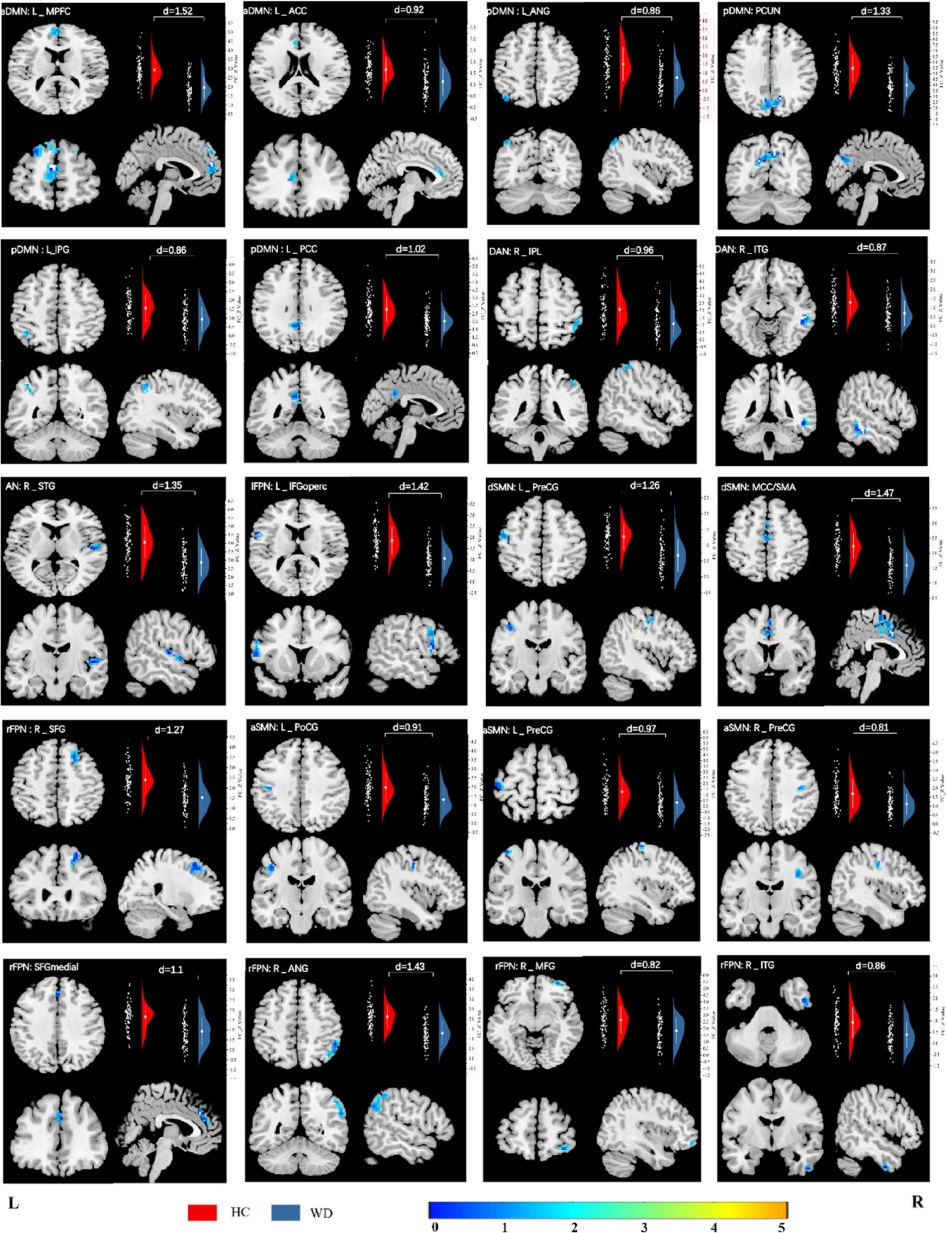
Group differences between WD and HC revealed by intra-network integrity analysis using template matching, including visualization of abnormal brain regions and violin plots. Cohen’s d was shown above each of the violin plots. The z values of the two-sample t-test are represented by colored bars. Abbreviations: HC, healthy control; WD, Wilson’s disease; L_MPFC, left medial prefrontal cortex; L_ACC, left anterior cingulate gyrus, L_ANG, left angular gyrus; PCUN, precuneus; L_IPG, left inferior parietal, but supramarginal and angular gyrus; L_PCC, left posterior cingulate gyrus, R_IPL, right inferior parietal lobule, R_ITG, right inferior temporal gyrus; R_STG, right superior temporal gyrus; L_IFGoperc, left inferior frontal gyrus, opercular part; L_PreCG, left precentral gyrus; MCC/SMA, middle cingulate gyrus/supplementary motor area; R_SFG, right superior frontal gyrus, dorsolateral; L_PoCG, left postcentral gyrus; SFGmedial, superior frontal gyrus, medial; R_ANG, right angular gyrus; R_MFG, right middle frontal gyrus

The abnormal RSNs identified were aDMN, pDMN, DAN, AN, lFPN, rFPN, dSMN, vSMN, between the HC and WD group (*P* < 0.05, cluster-level FWE correction). Compared with the controls, the patients with WD showed widespread lower connectivity, including the L_MPFC and the L_ACC within the aDMN, the left angular gyrus (L_ANG), the PCUN, the left inferior parietal, but supramarginal and angular gyrus (L_IPG) and the left posterior cingulate gyrus (L_PCC) within the pDMN, the right inferior parietal lobule (R_IPL) and the right inferior temporal gyrus (R_ITG) within the DAN, the right superior temporal gyrus (R_STG) within the AN, the left precentral gyrus (L_PreCG) and the middle cingulate gyrus/supplementary motor area (MCC/SMA) within the dSMN, the left postcentral gyrus (L_PoCG) and the bilateral PreCG within the vSMN, the left inferior frontal gyrus, opercular part (L_IFGoperc) within lFPN, the right superior frontal gyrus, dorsolateral (R_SFG), the superior frontal gyrus, medial (SFGmedial), the right angular gyrus ( R_ANG), the right middle frontal gyrus (R_MFG) and the R_ITG within the rFPN. The regions of the brain that displayed significantly altered intrinsic FC between the HC and WD groups were described in detail in Table 2 and Fig. 2.

**Table 2:** Brain regions of significant differences between the HC and WD groups in terms of FC in 8 RSNs

Brain region	RSNs	Cluster size (voxels)	Peak z-score	MNI Coordinates (x,y,z)
MCC/SMA	dSMN	151	5.51	3 9 39/3 -15 51
L_PreCG	dSMN	43	4.562	-42 -15 48
L_PoCG	vSMN	20	4.68	-43 -18 41
R_PreCG	vSMN	22	4.24	42 -14 41
L_PreCG	vSMN	17	4.69	-42 -24 66
R_ITG	rFPN	15	4.95	51 -3 -42
R_MFG	rFPN	16	4.31	33 57 -12
SFGmedial	rFPN	35	5.33	3 36 33
R_ANG	rFPN	98	4.7	54 -45 48
R_SFG	rFPN	88	5.11	27 24 54
PCUN	pDMN	117	5.59	12 -66 36
L_PCC	pDMN	45	4.66	-6 -51 30
L_IPG	pDMN	22	4.03	-39 -51 48
L_ANG	pDMN	23	4.16	-42 -69 48
L_IFGoperc	lFPN	63	6.55	-57 9 12
R_ITG	DAN	57	5.25	57 -36 -15
R_IPL	DAN	22	4.03	48 -39 60
R_STG	AN	70	5.48	57 -3 0
L_MPFC	aDMN	173	5.27	-6 57 26
L_ACC	aDMN	22	4.02	-6 36 12

**Fig. 3:**
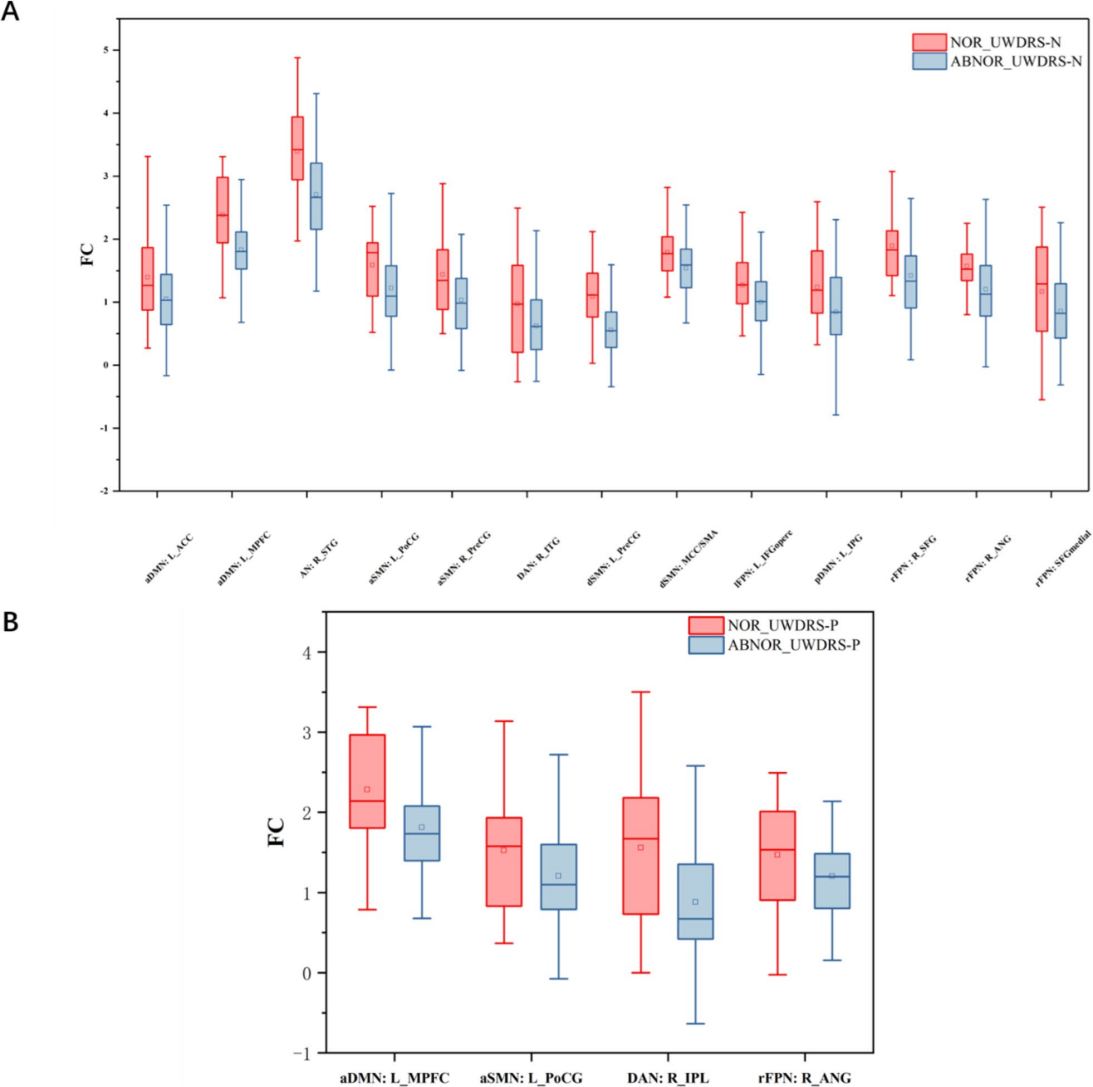
FC differences between WD patients with abnormal UWDRS scores and those with normal scores. Abbreviations: NOR_UWDRS-N, patients with normal UWDRS-N scores; ABNOR_UWDRS-N, patients with abnormal UWDRS-N scores; NOR_UWDRS-P, patients with normal UWDRS-P scores; ABNOR_UWDRS-P, patients with abnormal UWDRS-P scores

A two-sample t-test was conducted to compare the difference in FC between WD patients with abnormal UWDRS scores and those with normal scores. The analysis revealed that patients with abnormal scores exhibited significantly lower connectivity compared to those with normal scores. Please refer to Tables 3 and 4 and Fig. 3 for further information.

**Table 3:** Brain regions of significant differences between WD patients with abnormal UWDRS-N scores and those with normal scores

Brain region	NOR_UWDRS-N(*n* = 20)	ABNOR_UWDRS-N(*n* = 65)	*P* Value	T Value
dSMN: MCC/SMA	1.78 ± 0.42	1.53 ± 0.42	0.02	2.27
dSMN: L_PreCG	1.09 ± 0.53	0.55 ± 0.43	0.00	4.53
aSMN: L_PoCG	1.58 ± 0.57	1.22 ± 0.67	0.03	2.19
aSMN: R_PreCG	1.43 ± 0.71	1.03 ± 0.51	0.01	2.80
rFPN: SFGmedial	1.36 ± 0.84	0.86 ± 0.62	0.04	1.91
rFPN: R_ANG	1.57 ± 0.46	1.21 ± 0.57	0.01	2.61
rFPN: R_SFG	1.89 ± 0.54	1.41 ± 0.65	0.01	2.97
pDMN: L_IPG	1.23 ± 0.64	0.84 ± 0.68	0.02	2.30
lFPN: L_IFGoperc	1.27 ± 0.51	0.99 ± 0.50	0.03	2.11
DAN: R_ITG	0.97 ± 0.79	0.62 ± 0.64	0.04	2.01
AN: R_STG	3.39 ± 0.71	2.70 ± 0.77	0.00	3.53
aDMN: L_MPFC	2.38 ± 0.66	1.80 ± 0.55	0.00	3.69
aDMN: L_ACC	1.39 ± 0.75	1.05 ± 0.53	0.02	2.29

**Table 4:** Brain regions of significant differences between WD patients with abnormal UWDRS-P scores and those with normal scores

Brain region	NOR_UWDRS-P(*n* = 27)	ABNOR_UWDRS-P(*n* = 58)	*P* Value	T Value
aSMN: L_PoCG	1.36 ± 0.58	1.04 ± 0.51	0.01	2.57
rFPN: R_ANG	1.46 ± 0.63	1.21 ± 0.53	0.04	2.01
DAN: R_IPL	1.56 ± 0.98	0.88 ± 0.80	0.00	3.38
aDMN: L_MPFC	2.28 ± 0.63	1.81 ± 0.56	0.00	3.46

### Aberrant inter-network FC

**Fig. 4:**
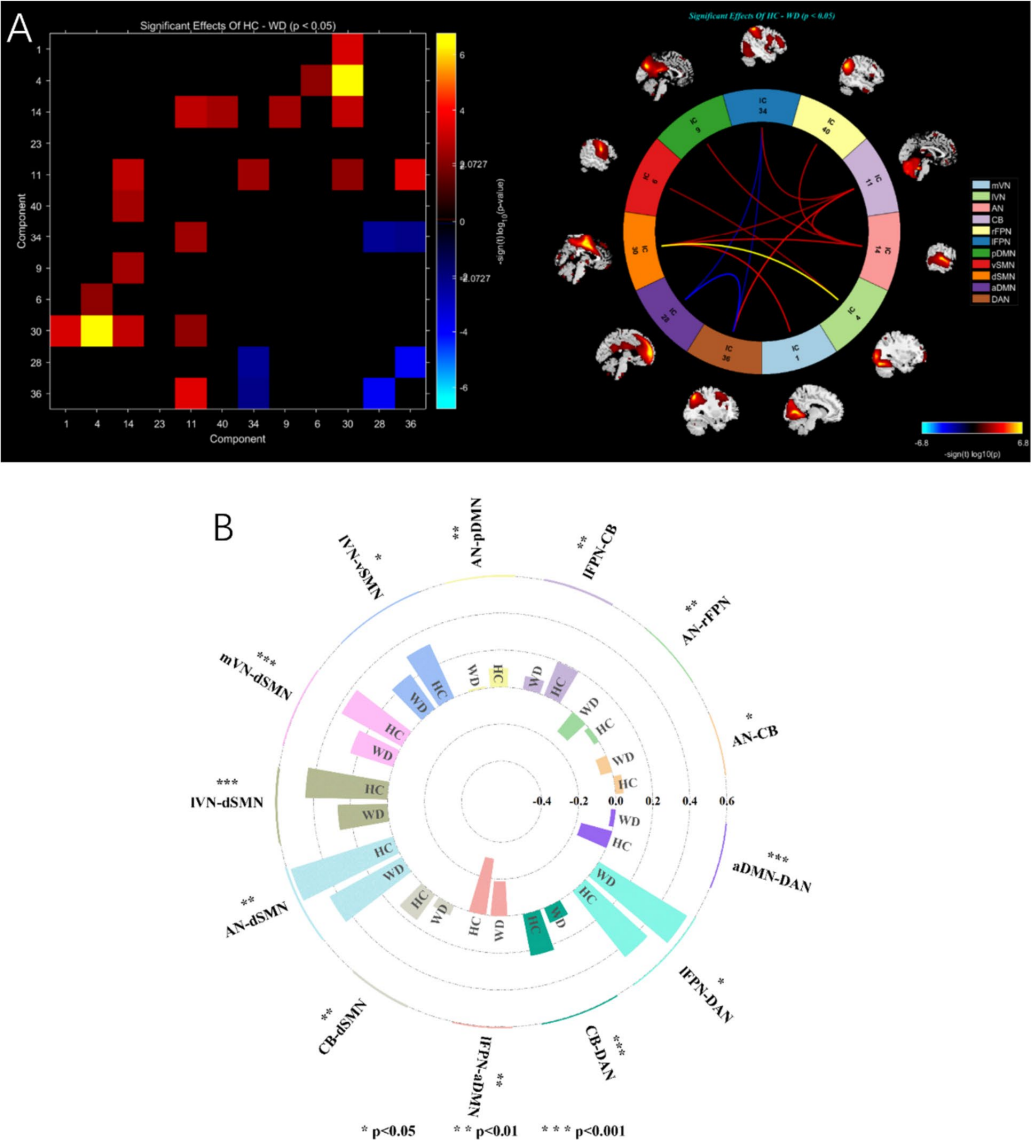
Significant differences in inter-network FC between patients with WD compared with healthy comparisons were represented with correlation matrix and circle diagrams (**A**) and the mean value of the inter-network FC signal (**B**). Abbreviations: mVN, medial visual network; lVN, lateral visual network; AN, auditory network; aDMN, anterior default mode network; pDMN, posterior default mode network; lFPN, left frontoparietal network; rFPN right frontoparietal network; CB cerebellum network; vSMN, ventral somatomotor network; dSMN, dorsal somatomotor network; DAN, dorsal attention network

Compared with HC, the FC was significantly increased between the aDMN and DAN (*P* < 0.001, *d* = 0.57), lFPN and DAN (*P* < 0.05, *d* = 0.40), as well as between lFPN and aDMN (*P* < 0.01, *d* = 0.44) in patients with WD. Moreover, it was observed that the FC in WD group was significantly decreased between AN and CB (*P* < 0.05, *d* = 0.40), AN and rFPN (*P* < 0.01, *d* = 0.48), lFPN and CB (*P* < 0.01, *d* = 0.46), AN and pDMN (*P* < 0.01, *d* = 0.43), lVN and vSMN (*P* < 0.05, *d* = 0.37), mVN and dSMN (*P* < 0.001, *d* = 0.50), lVN and dSMN (*P* < 0.001, *d* = 0.76), AN and dSMN (*P* < 0.01, *d* = 0.46), CB and dSMN (*P* < 0.01, *d* = 0.41), as well as between CB and DAN (*P* < 0.001, *d* = 0.56) (Fig. 4 A and B).

### Correlation between the FC and clinical characteristics

**Fig. 5:**
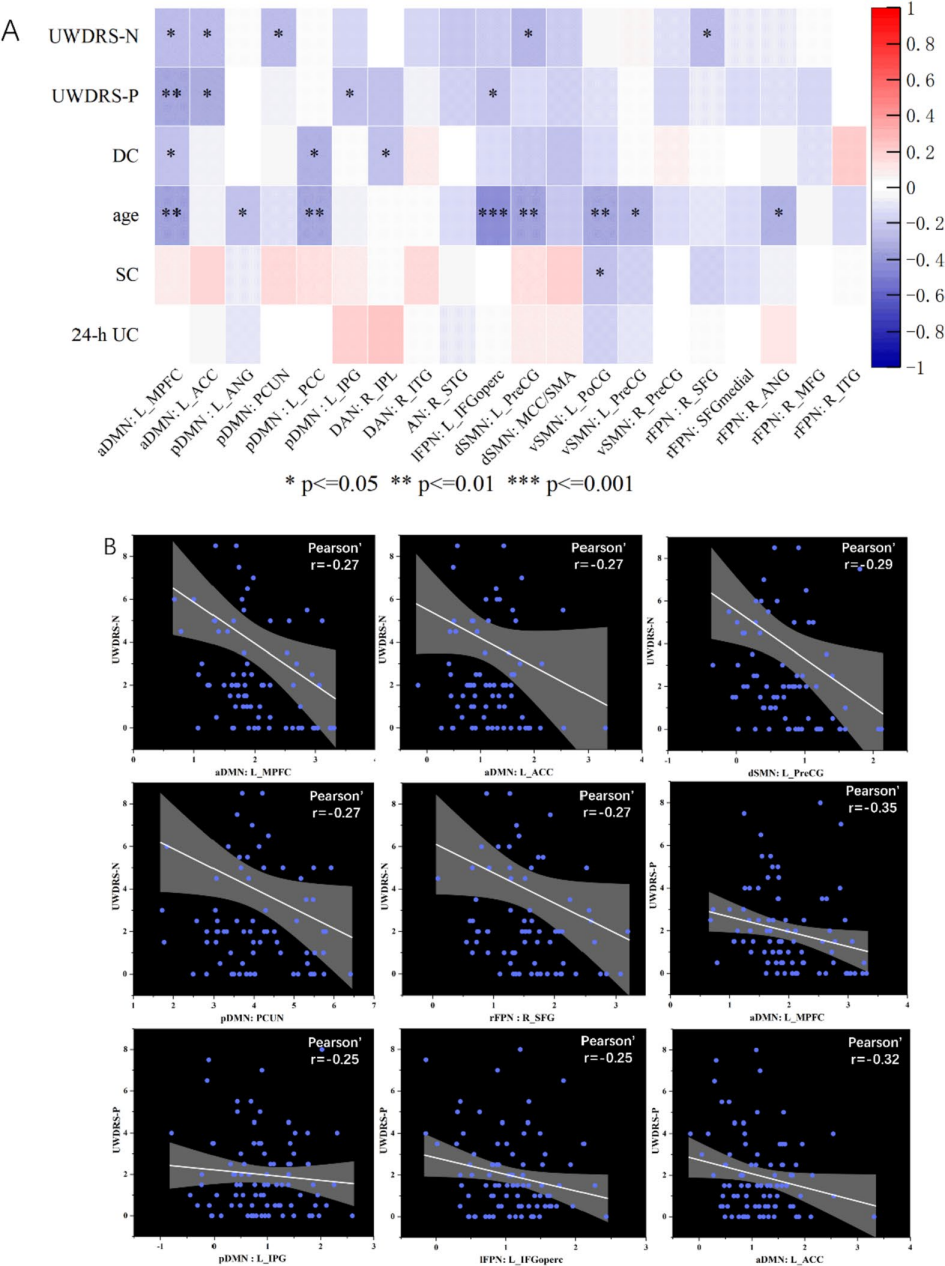
Correlations between clinical characteristics and FC values of abnormal brain regions. Results of correlation analysis were presented as a heat map of the correlation matrix (**A**) and correlation diagram (**B**). Red indicates positive correlation and blue indicates negative correlation

Significant correlations between intra-network FCs and the clinical assessments were shown as a heat map of the correlation matrix (Fig. 5A), and the detailed information was displayed in Fig. 5B. The UWDRS-N score was negatively correlated with the L_MPFC, L_PCC (*r* = -0.27, *p* < 0.05) within the aDMN, the PCUN (*r* = -0.27, *p* < 0.05) within the pDMN, the L_PreCG (*r* = -0.29, *p* < 0.05) within dSMN and the R_SFG (*r* = -0.27, *p* < 0.05) within rFPN. The UWDRS-P score was negatively correlated with the L_MPFC (*r* = -0.35, *p* < 0.01), the L_ACC (*r* = -0.32, *p* < 0.05) within aDMN, the L_IPG (*r* = -0.25, *p* < 0.05) within pDMN, the L_IFGoperc (*r* = -0.25, *p* < 0.05) within lFPN. The DC was negatively correlated with the L_MPFC (*r* = -0.26, *p* < 0.05) within aDMN, the L_PCC (*r* = -0.31, *p* < 0.05) within pDMN, and the R_IPL (*r* = -0.25, *p* < 0.05) within DAN. The age was negatively correlated with the L_MPFC (*r* = -0.36, *p* < 0.01) within aDMN, the L_ANG (*r* = -0.26, *p* < 0.05) and the L_PCC (*r* = -0.35, *p* < 0.01) within pDMN, the L_IFGoperc (*r* = -0.45, *p* < 0.001) within lFPN, the L_PreCG (*r* = -0.38, *p* < 0.01) within dSMN, the L_PoCG (*r* = -0.36, *p* < 0.01) and L_PreCG (*r* = -0.31, *p* < 0.05) within vSMN, the R_ANG (*r* = -0.30, *p* < 0.05) within rFPN. The SC was negatively correlated with the L_PoCG (*r* = -0.30, *p* < 0.05) within vSMN. In terms of inter-network, there was no significant correlation between changes in FC of brain networks and clinical assessments.

## Discussion

A large cohort study investigated the correlation between MRI brain pathology and the MRI scale with disease form and neurological severity. The findings revealed that the semiquantitative brain MRI scale showed a strong correlation with the MRI changes and UWDRS [11]. Smolinski [34] discovered that the rate of brain atrophy in individuals with neurological WD was significantly higher. This degeneration was found to be linked to the advancement of neurological decline and the levels of NfL in the blood at the beginning of the study. To date, little attention has been paid to concentrate on the variations in network connectivity in the brain of individual with WD. In the current study, we employed rs-fMRI with ICA analysis to investigate alterations in FCs of intra- and inter-networks in brain, and further explored potential associations with clinical measures based on an ample sample size. The findings confirmed alteration in both the intra-network and inter-network FC patterns. Compared with the controls, WD patients showed widespread lower connectivity within RSNs and higher connectivity between DMN, DAN and FPN. These findings recommend that WD is probably associated with cortical network connectivity abnormalities, imparting perception into the pathophysiology of this complicated disorder. Moreover, we observed significant correlations between age with decreased FC in multi-brain areas, involved L_MPFC, L_ANG, L_PCC, L_IFGoperc, L_PoCG and L_PreCG, R_ANG, which also provided evidence that WD is an ongoing and progressive condition.

### Altered FCs in higher-order cognitive control networks

It was reported that DMN, FPN, DAN and SN networks play central roles in cognitive control. They show complicated patterns of context-dependent dynamic interactions amongst each other [35]. Dynamic integrative between-network conversation is fundamental for efficient cognitive manipulate and adaptive behaviors [36]. The DMN, as a baseline state of the human brain related with consciousness maintenance, has been regarded to be concerned in the integration of primary perception and high cognitive processing [37, 38]. In our study, FC alterations within the DMN has appeared in WD patients. Compared to HC, WD patients showed lower connectivity in the L_MPFC, L_ACC/PCC and PCUN. Furthermore, the decreased FCs in the L_MPFC, L_ACC, PCUN were negatively correlated with the UWDRS-N, and the decreased FCs in the L_MPFC, PCUN were negatively correlated with the UWDRS-P. It suggested that the reduced FC in the DMN network would possibly lead to neurological signs and psychiatric symptoms in WD patient. The findings of DMN exhibited to be in accordance with Han [22], who reported that WD patients had altered DMN’s FC and lower local and global network efficiency compared with normal controls. As a key node of the DMN, the MPFC is cautioned to be one of the highest associative facilities in the frontal lobe taking part in cognitive strategies and auto biographical retrieval, which can coordinate of mental processes and action in accordance with current goals and future plans [39]. Several studies have demonstrated a contribution from the PCUN, an associative cortex involved in highly integrated cognitive tasks [40]. DAN, an antagonist network of the DMN [41], responds via directing bottom-up attention to salient environmental stimulation when the brain is engaged in attentionally demanding cognitive processes [42]. The FPN is a practical hub that influences whole-brain conversation to meet multiple demand tasks, with areas of the lateral frontal cortex, dorsomedial frontal cortex, and parietal cortex involved [43, 44]. These areas within the FPN are activated for the duration of duties requiring cognitive management or executive function [45]. In this study, lowered FC in DNA and bilateral FPNs had been detected, in particular the rFPN(i.e. ITG, MFG). Previous studies indicated that cognitive and psychiatric problems in WD patients might result from the joint abnormal functions of ITG, MFG [46, 47]. Also, we observed that reduced FC in the FPN network used to be negatively correlated with the UWDRS-N. Studies focusing on neuropsychological impairments in WD have demonstrated that patients with WD showing neurological signs present significant deficits in a wide range of cognitive domains [48, 49], and sufferers recruited in this study show impairments in executive function. It suggested that disruption in DMN, DAN, FPN networks and integrative conversations between them, which involve the failure of higher-order cognitive processes, might provide a neurophysiological account of the maladaptive daily behaviors of WD patients.

Interestingly, we additionally discovered that the patients with WD exhibited significantly stronger FC between the lFPN and aDMN, between the DAN and aDMN, and between the lFPN and DAN compared to HC (Fig. 3A). In a previous study, Fan showed significantly enhanced coupling between the DMN and the FPN and a negative correlation between functional connectivity and tic severity in the DMN in patients with Tourette’s disorder. They speculated that the compensation for altered FC within these two networks might be a compensatory mechanism to overcome motor tics [50]. In addition, significantly enhanced FCs between the rFPN, aDMN, and the attention network (ATN) were exhibited as well in patients with idiopathic generalized epilepsy [51]. The normative pattern of anticorrelation between DMN and ATN, a binding mechanism between an introspective and an extrospective attentional orientation, is required to keep the brain's intrinsic functional organization dynamically balanced [52, 53]. In our study, the results might suggest that the shifting balance within these networks could be associated with deficits in cognitive control and attention in patients with WD. In WD patients, chain alterations of the inter-network useful coupling in attention and high-order cognitive manipulation networks were observed. These findings were especially essential for perception in the pathophysiology of WD due to the fact that cortical reorganization is a common mechanism of compensation for cortical injury, which disrupts the associated networks that guide cognitive behavior.

### Altered FCs in motor control networks

It was known that the cortex of the frontal and parietal lobes performs an essential function in improving movement as direct and indirect comments channels for facts processing [54]. In the current study, we found that the FC in the SMA and the primary sensorimotor cortex (S1/M1) (i.e. PoCG / PreCG) within SMN and premotor cortex (PMC, i.e. SFG and MFG) within FPN was diminished in patients with WD. These regions were important nodes in the flow of information from the cortex-SMA-PMC-anterior horn of the spinal cord, which contributes to the control of movement and the regulation of posture [55]. Postural tremor, dysphonia and dystonia are frequent neurological manifestation in patients with WD [56, 57]. The primary sensorimotor cortex (S1/M1) has been proven to be involved in WD postural tremor generation [58]. Functional variations in the sensorimotor network have been identified in patients with spasmodic dysphonia, a kind of laryngeal dystonia [59]. The PMC and SMA generate the programs for precise posture and movement control. The SMA receives information from the external environment and projects the efferent information to the motor areas after the information has been integrated and processed [60]. PMC may transmit movement programs to the primary motor cortex, which would then transmit movement commands via the corticospinal tract. Besides, evidence suggests that the cerebellum plays a key role in postural tremor [61]. In our study, decreased functional connectivity occurred between the cerebellum and many of the other networks (e.g. SMN, lFPN, DAN and AN), even though the alteration in functional connectivity within the cerebellum was not found. Cerebellar involvement in dystonic tremor involved widespread functional changes [62]. The cerebellum is involved in processing tremor-related afferents, and it was confirmed that the abnormality in cerebello-thalamo-cortical motor pathway is involved in tremor generation [63]. Also, in patients with essential tremor, it was showed decreased functional connectivity between the cerebellar network and the sensorimotor network [64], which exhibited to be in accordance with our findings. Thus, it could be speculated that the occurrence of neurological manifestation (e.g. postural tremor, dysphonia and dystonia) in WD may be associated with the disruption of the cortex-SMA-PMC-anterior horn loop or cerebello-thalamo-cortical motor pathway or disruptions between networks. It was demonstrated that PSA-DBS has remarkable efficacy in effectively managing both the tremor and dystonia associated with WD. Results in this study may serve as a valuable theoretical foundation for future treatments, including Low's reported case of PSA-DBS in Wilson's disease [56].

### Limitations and conclusion

In this study, we investigated changes in large-scale functional networks in WD patients using a data-driven approach of ICA. The results obtained from the network will depend on the data being extracted. Despite having a sufficient sample size, the basal ganglia network [65], which is typically affected in WD, was not detected. As we know, the basal ganglia network, which is most commonly damaged in WD, was not detected, although the sample size was sufficient. However, in future studies, we could enhance our understanding of the basal ganglia network by constructing and analyzing it using graph theory. Besides, the resting-state functional connectivity patterns of WD patients were detected in this study, structural networks in the brain may provide complementary information to the current study.

To conclude, we have provided evidence that WD is a disease with widespread dysfunctional connectivity in resting networks in brain, leading to neurological features and psychiatric symptoms (e.g. higher-order cognitive control and motor control impairments). The altered intra- and inter-network in the brain may be the neural underpinnings for the neuropathological symptoms and the process of injury compensation in WD patients.

## Data Availability

The datasets used in the current study are available from the corresponding author on reasonable request.

## Abbreviations

AN: Auditory network
ATN: Attention network
CB: Cerebellum network
DAN: Dorsal attention network
DC: Course of disease
DMN: Default mode network
FA: Flip angle
FCs: Functional connections
FDR: False discovery rate
FOV: Field of view
FPN: Frontoparietal network
HC: Healthy control
ICA: Independent component analysis
ICs: Independent components
KF: Kayser-Fleischer
SC: Serum ceruloplasmin
L_ACC: Left anterior cingulate gyrus
L_ANG: Left angular gyrus
L_IFGoperc: Left inferior frontal gyrus, opercular part
L_IPG: Left inferior parietal, but supramarginal and angular gyrus
L_ MPFC: Left medial prefrontal cortex
MCC/SMA: Middle cingulate gyrus/supplementary motor area
mVN: Medial visual network
PCA: Principal component analysis
L_PCC: Left posterior cingulate gyrus
L_PoCG: Left postcentral gyrus
L_PreCG: Left precentral gyrus
NOR_UWDRS-N: Patients with normal UWDRS-N scores
ABNOR_UWDRS-N: patients with abnormal UWDRS-N scores
NOR_UWDRS-P: Patients with normal UWDRS-P scores
ABNOR_UWDRS-P: Patients with abnormal UWDRS-P scores
PCUN: Precuneus
PCC: Posterior cingulate cortex
PMC: Premotor cortex
R_ANG: Right angular gyrus
R_FPN: Right frontoparietal network
R_MFG: Right middle frontal gyrus
R_IPL: Right inferior parietal lobule
R_ITG: Right inferior temporal gyrus
R_SFG: Right superior frontal gyrus, dorsolateral
R_STG: Right superior temporal gyrus
rs-fMRI: Resting-state functional magnetic resonance imaging
RSNs: Resting-state networks
SFGmedial: Superior frontal gyrus, medial
SMN: Somatomotor network
SN: Salience network
TE: Echo time
TR: Repetition time
UWDRS-N: The Unified Wilson’s Disease Rating Scale-neurological characteristic examination
UWDRS-P: The Unified Wilson’s Disease Rating Scale-psychiatric symptoms examination
WD: Wilson’s disease
24-h UC: 24-H urinary copper
